# Pharmacological profiles and mechanism of action of remimazolam for ICU sedation

**DOI:** 10.1186/s40560-026-00858-7

**Published:** 2026-02-11

**Authors:** Kenichi Masui

**Affiliations:** Department of Anesthesiology and Intensive Care, Yokohama City University, Fukuura 3-9, Kanazawa-Ku, Yokohama, 236-0004 Japan

**Keywords:** Remimazolam, Benzodiazepine, Sedation, Intensive care unit, Pharmacokinetics, Context-sensitive decrement time, Short-acting, Accumulation, Flumazenil

## Abstract

**Background:**

Remimazolam, a benzodiazepine, was first approved in 2020 for general anesthesia and procedural sedation but has not for sedation in the intensive care units. As other sedatives used in intensive care units have disadvantages such as propofol infusion syndrome and hypotension, an alternative sedative would be desired.

**Main body:**

Remimazolam was developed as a soft drug that is designed to be rapidly metabolized to the inactive chemical, CNS7054. In humans, it is mainly metabolized by the non-specific esterase, carboxylesterase 1, in the liver, not in the blood, unlike the ultrashort-acting soft drug remifentanil. Remimazolam is a short-acting drug. The context-sensitive decrement time, which describes how the accumulated drug affects the decay curve of the drug concentration after its infusion ends, of remimazolam is similar to that of propofol. Remimazolam binds the benzodiazepine site of the gamma-aminobutyric acid type A receptors, and does not directly activate the gamma-aminobutyric acid type A receptors. Benzodiazepines are not effective in the absence of gamma-aminobutyric acid. Although hypotension appears less frequent, it can occur during remimazolam administration. Delayed emergence can occur after remimazolam infusion due to its accumulation. To prevent delayed emergence, excessive doses should be avoided. A dose of the competitive antagonist flumazenil can cause re-sedation after administration. The impact of delirium after prolonged sedation is unknown. The remimazolam concentration at steady-state is approximately 10% higher in males than in females, > 20% in obese patients than in normal weight patients, and approximately 20% higher in American Society of Anesthesiologists physical status III/IV patients than in I/II patients. The infusion rate should may be reduced in these patients. A study has shown that the required dose is higher in children than in adults.

**Conclusions:**

Remimazolam could be an alternative sedative in intensive care units. Because remimazolam is short-acting, it accumulates during prolonged infusions. Infusion rate should be titrated based on patient characteristics, including sex, body mass index, and physical status. Benzodiazepine tolerance should be considered before and during remimazolam infusion. Similar to other sedatives, concurrent use of additional hypnotics and analgesics may help maintain the desired sedation in intensive care units.

## Background

Propofol, midazolam, and dexmedetomidine are widely used for sedation in the intensive care unit (ICU) [1]. These drugs have advantages such as being short-acting [2], having less depressant effects on hemodynamics [3], and having a lower risk of delirium [1], whereas they also have disadvantages such as propofol infusion syndrome [4, 5], slow decrease in drug concentration [2], not being suitable for deep sedation [6], and bradycardia, and hypotension [7].

Remimazolam besylate is a novel benzodiazepine sedative/anesthetic, which was first approved in Japan in 2020 as an anesthetic [8] and was approved as an anesthetic and sedative for procedural sedation in various countries. As the pharmacological characteristics of short-acting benzodiazepines and standard sedatives for ICU sedation have limitations, remimazolam is expected to be an alternative because it can achieve deep sedation, has a low risk of hemodynamic instability, and does not cause propofol infusion syndrome. Remimazolam may be a suitable sedative not only for adults but also for children, reducing the incidence of these side effects. The presence of an antagonist, flumazenil, might enhance the value of remimazolam in ICU sedation. For example, this antagonist may help evaluate patient consciousness during remimazolam sedation or aid in treating airway difficulties after remimazolam.

However, there are some issues with using remimazolam for ICU sedation. First, remimazolam is not currently approved for ICU sedation, and regulatory submission was not pursued after the clinical trial was terminated in 2013 due to unexpectedly high concentrations during sedation [9]. This raises concern that remimazolam might not be appropriate for ICU sedation. Second, remimazolam tolerance has been reported. Several case reports have described tolerance to remimazolam in long-term benzodiazepine users or patients with benzodiazepine dependency [10–12]. In some cases, remimazolam might cause acute tolerance during administration [13].

The purpose of this review is to explain the pharmacological profiles and mechanism of action of remimazolam, which may potentially improve ICU sedation, and to propose a dosing strategy for ICU sedation.

### Chemical structure of remimazolam designed as a soft drug

Remimazolam was designed as a soft drug, which is rapidly metabolized to inactive chemical substances. Similar to an ultrashort-acting opioid, remifentanil, remimazolam contains an ester bond. While remifentanil is metabolized by nonspecific esterase in the blood, remimazolam is primarily metabolized by carboxylesterase 1 to CNS7054 in the liver [14]. The affinity of CNS7054 is 410-fold less than that of remimazolam. Although carboxyl esterase 1 is also a nonspecific esterase, it does not exist in the human blood [15]. One article has shown that venous plasma remimazolam concentrations were not lower than arterial plasma remimazolam concentrations during a constant-rate infusion [16], indicating remimazolam is minimally or not metabolized in the blood. This difference in the site of metabolism may be a reason why remimazolam is an ultrashort-acting but short-acting drug.

### Pharmacokinetic profile: not ultra-short acting but short acting

**Fig. 1:**
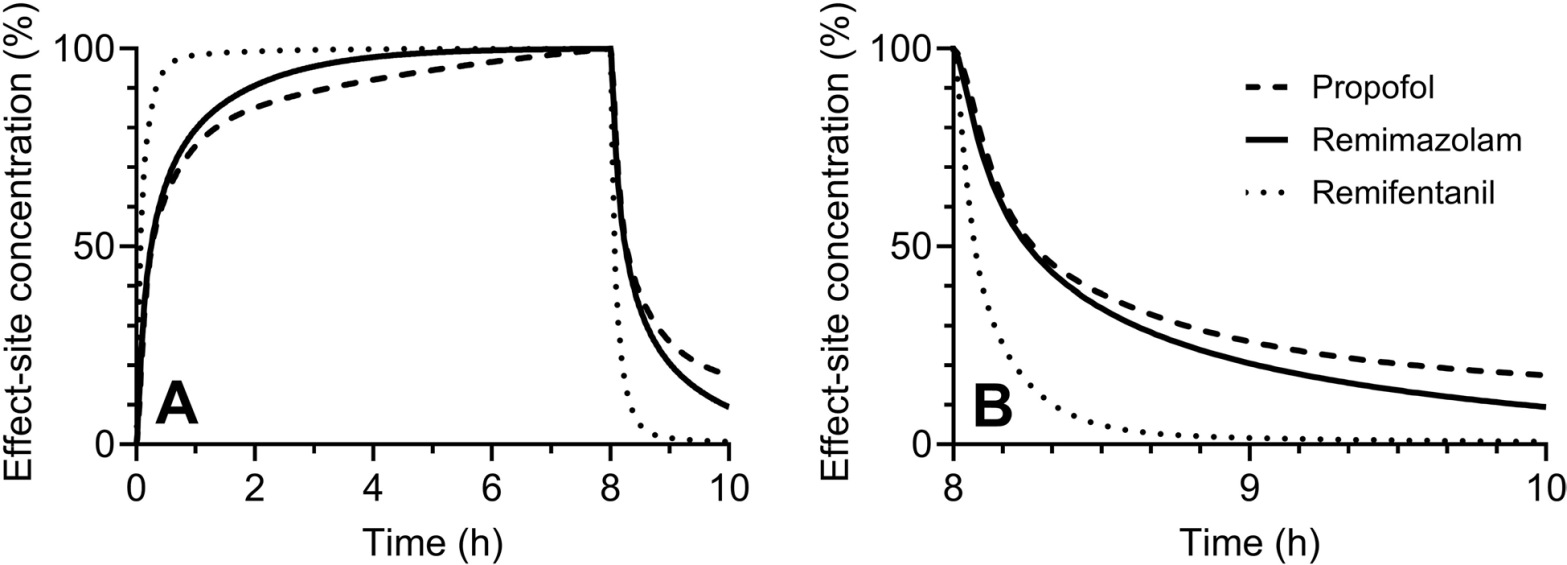
Time course of effect-site concentrations of propofol, remimazolam, and remifentanil during **A** and after **A**,** B** a constant rate infusion for 8 h. The effect-site concentrations at 8 h after the start of the infusion are defined as 100%. The time course of remifentanil after termination of the infusions **B** is similar to that of propofol for the first 30 min, whereas it is different from that of remifentanil. The Marsh model [18] was used for these calculations

Many articles say remimazolam is an ultrashort-acting agent. However, this term would not reflect its pharmacokinetic characteristics. As shown in Fig. 1, the decay curve’s shape of remimazolam is similar to that of propofol but not to that of remifentanil after a continuous infusion for several hours. Propofol and remifentanil are called short-acting and ultrashort-acting, respectively. Accordingly, it is better to say remimazolam as a short-acting but not ultrashort-acting drug because the term “short-acting” describes its characteristic well compared to recent standard anesthetics and opioids.

Note that midazolam, a conventional benzodiazepine, is also called a short-acting drug. As the duration of remimazolam’s effect is shorter than that of midazolam, this difference in duration may be a reason why remimazolam is called an ultrashort-acting.

To understand the decay curves and accumulation of remimazolam, context-sensitive decrement time is helpful.

### Context-sensitive decrement time: understanding decay time and accumulation

Context-sensitive decrement time (CSDT) is a concept used to describe how accumulated drug affects the decay curve of the drug concentration after its infusion ends. Originally, context-sensitive half-time (CSHT) was developed for the same concept [2].

**Fig. 2:**
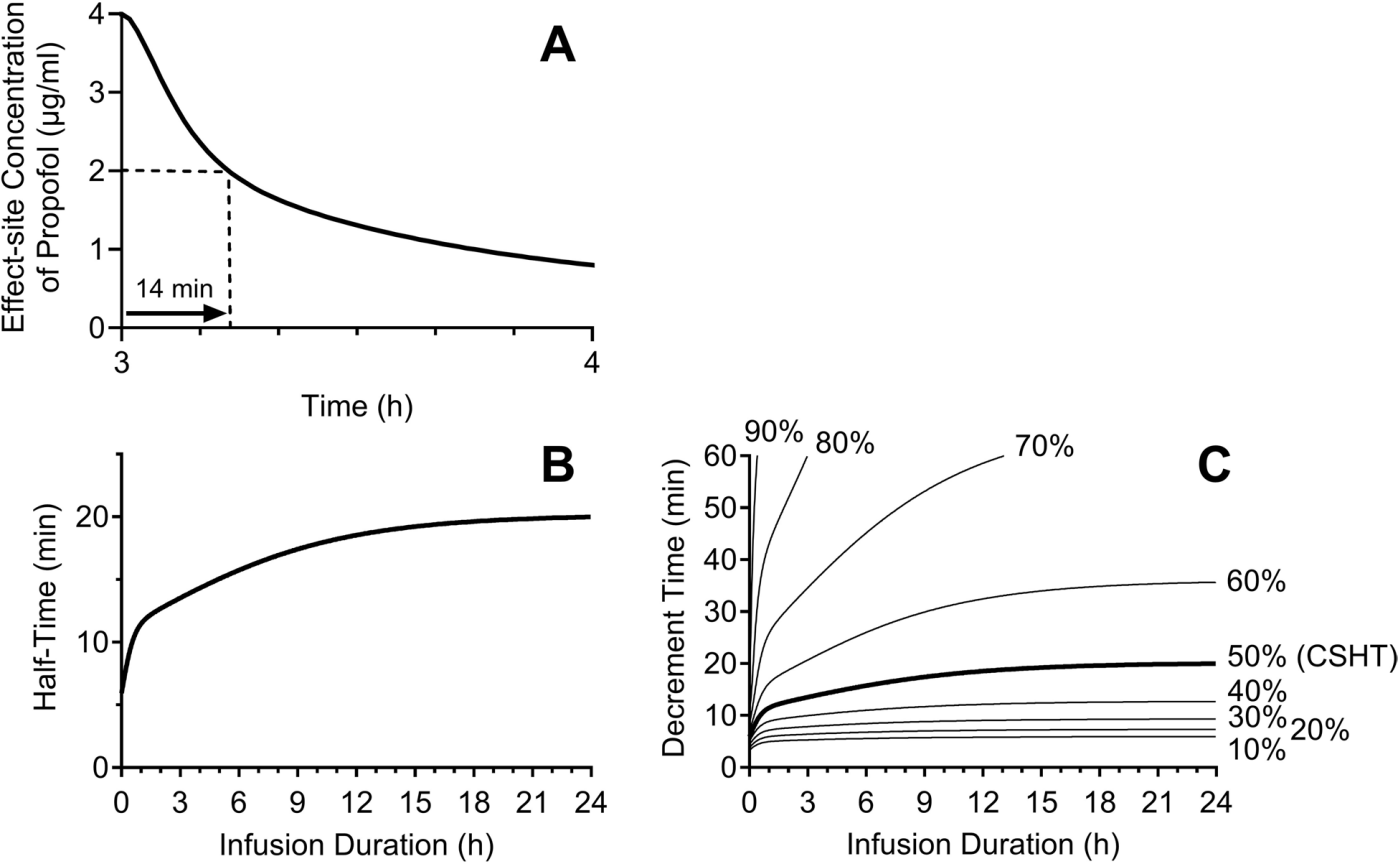
**A** Time course of the effect-site concentration of propofol after the termination of 3 h using target-controlled infusion at 4 µg/mL. After the termination, the “decrement time” of propofol concentration from 4 µg/mL to 2 µg/mL is 14 min. **B** Context-sensitive half-time (CSHT) of propofol. When the infusion duration is 3 h, the half-time is 14 min as shown in Fig A. **C** Context-sensitive decrement times (CSDTs) of propofol. The Marsh model [18] was used for these calculations

The following example explains how to calculate the CSHT using propofol administered for 3 h. Propofol is administered virtually at a constant plasma concentration of 4 µg/mL using the Marsh pharmacokinetic model [17], similar to a plasma-targeted target-controlled infusion (TCI) set to 4 µg/mL, for 3 h using a pharmacokinetic model of the drug. A simulation shows the decay curve of plasma propofol concentration after the 3-h infusion (Fig. 2A). The half-time for a 3-h infusion is calculated as the time from the end of the continuous infusion, maintaining the plasma concentration at 4 µg/mL, to the time at which the plasma concentration decreases to 2 µg/mL (14 min, Fig. 2A). The entire curve of CSHT for propofol is described in Fig. 2B, where the x-axis represents the infusion time of propofol at a constant concentration, and the y-axis shows the half-time after the end of propofol infusion. This ‘context-sensitive’ half-time curve includes the half-time, 14 min, for a 3-h infusion of propofol shown in Fig. 2A. The CSHT curve clearly indicates that increasing the infusion time, i.e., accumulating more propofol, leads to a longer half-time. The ‘context-sensitive’ half-time indicates that the half-time is affected by the drug infusion history.

CSHT is used to understand the decay curve of drug concentration over hours after an infusion, as well as to predict the time from the end of infusion to when the drug’s effect disappears. For these purposes, the original CSHT has two limitations. The first is that the effect of some drugs, such as anesthetics, is determined by their effect-site concentration (Ce), not by their plasma concentration. Therefore, a CSHT for Ce is more useful for predicting the duration until the loss of the drug effect than the CSHT for plasma concentration (Cp). The second is that the half-time is not often equal to the time required for the drug effect to disappear. When a drug is administered at 4 times the maximal concentration when the effect is absent, a 75% decrease, not a 50% decrease, is required for the drug effect to disappear. In another example, when propofol is administered at 4 µg/mL using TCI in a patient who will recover from anesthesia at 1 µg/mL, a 75% decrement time, not a half-time (a 50% decrease time), is needed for the disappearance of the propofol effect, loss of consciousness, after the end of propofol infusion.

CSDTs in Fig. 2C represent various decrement times (10% to 90% decrement times) of the propofol Ce. For example, after propofol TCI of propofol at 3 µg/mL for 6 h, 40% decrement time, 50% decrement time (same as half-time), and 60% decrement time, i.e., the time from the termination of propofol TCI at 3 µg/mL to the time when the propofol Ce decreases to 1.8, 1.5, and 1.2 µg/mL, respectively, are 11, 16, and 26 min, respectively (Fig. 2C).

**Fig. 3:**
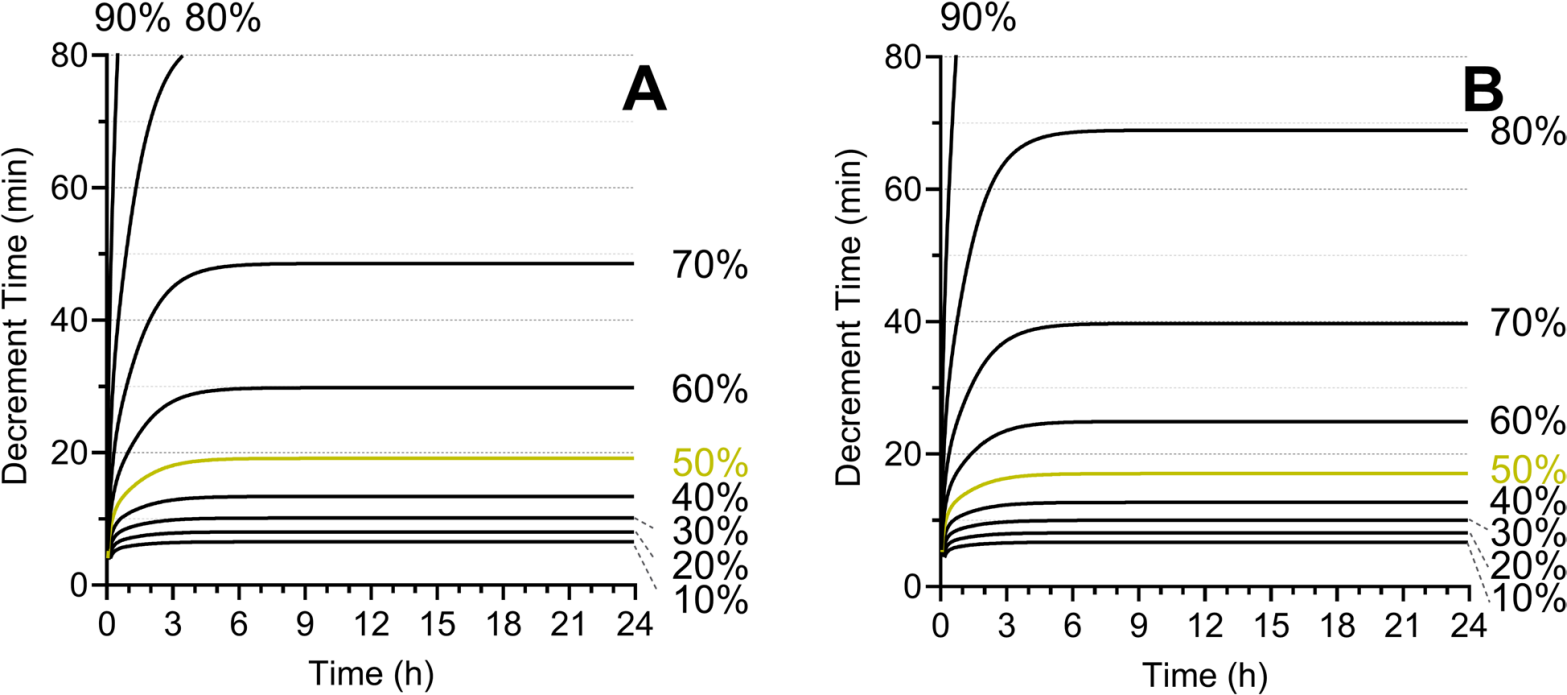
Context-sensitive decrement time of remimazolam in a male **A** or female **B** patient (55 years, American Society of Anesthesiologists physical status III, 70 kg, and 170 cm)

**Fig. 4:**
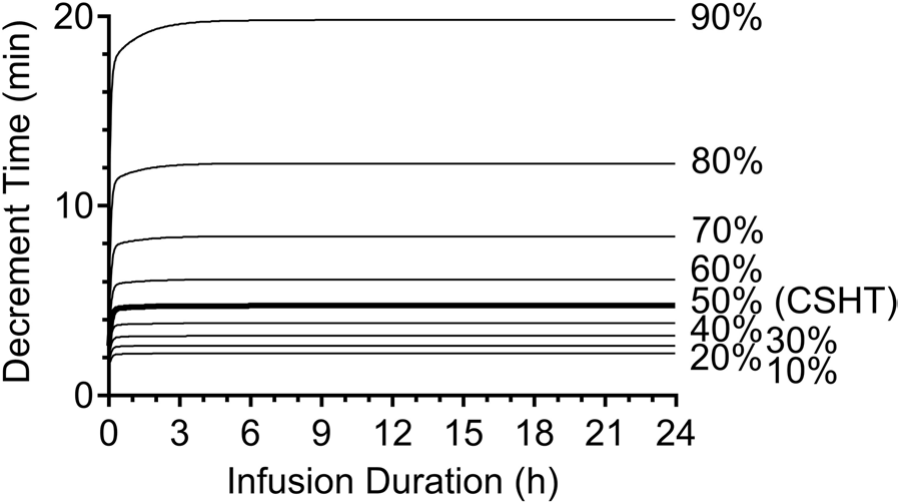
Context-sensitive decrement time of remifentanil

Figure 3A, B show the CSDTs for remimazolam Ce for Anesthesiologists physical status (ASA-PS) III/IV male and female patients, respectively, using the Masui pharmacokinetic model [18, 19], which indicates sex influences the pharmacokinetics of remimazolam. When comparing the CSDTs for remimazolam Ce (Figs. 3A, B) and the CSDTs for propofol Ce (Fig. 2C), both show similar decrement times. On the other hand, the CSDTs for remimazolam Ce (Fig. 3B) and remifentanil Ce (Fig. 4) using the Minto pharmacokinetic model [20] differ in their decrement times. These CSDT curves support that remimazolam is short-acting but not ultra-short-acting.

As shown in Fig. 3, remimazolam accumulates during a prolonged infusion, similar to propofol. For example, after remimazolam was infused at 1 µg/mL (this concentration is nearly equivalent to that achieved when administering remimazolam at 1 mg/kg/h) for 12 h in a female patient, who was 55 years, ASA-PS III, 70 kg, and 170 cm, the infusion was terminated. If this patient wakes up at 0.2 or 0.1 µg/mL of remimazolam, it will take 69 or 134 min from the termination of the remimazolam infusion to become awake. In other words, an overdose of remimazolam for prolonged ICU sedation might result in persisting sedation for > 120 min after discontinuation of an infusion.

### Mechanism of action

**Fig. 5:**
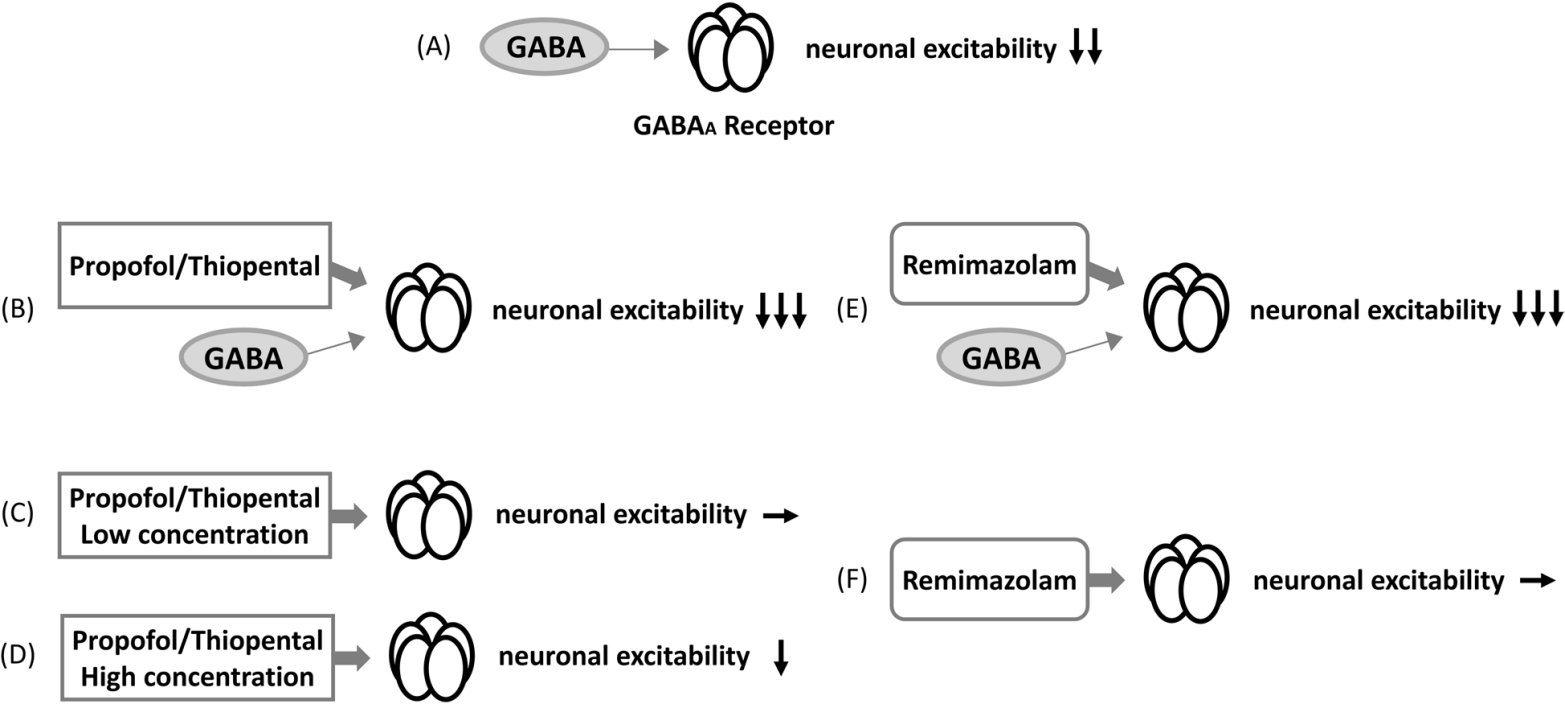
Influence of Sedatives on Neuronal Excitability. The presence of gamma-aminobutyric acid (GABA) binds to the GABA_A_ receptor, which decreases neuronal excitability **A**. Propofol and thiopental also bind to the GABA_A_ receptor, thereby enhancing the GABA-induced decrement in neuronal excitability **B**. A low dose of propofol/thiopental does not directly reduce neuronal excitability **C**, whereas a high dose of propofol/thiopental does directly **D**. Remimazolam also binds to the GABAA receptor, enhancing the GABA-induced decrease in neuronal excitability **E**. However, remimazolam has no direct impact on neuronal excitability **F**

Remimazolam binds to the benzodiazepine site of the gamma-aminobutyric acid type A (GABA_A_) receptor, which was activated by GABA and GABA_A_ receptor agonists. An article explains that mechanisms of action differ between benzodiazepines and propofol/thiopental as follows [21]. Lower concentrations of propofol/thiopental enhance the effect of GABA, while higher concentrations of propofol/thiopental directly activate the GABA_A_ receptor (Fig. 5). Additionally, propofol and some barbiturates decrease the extent of desensitization. On the contrary, benzodiazepines do not directly activate the GABA_A_ receptor. In other words, benzodiazepines are not effective in the absence of GABA [21, 22]. Additionally, benzodiazepines have no effect of desensitization under the saturating concentrations of GABA.

### Adverse event: hypotension

Randomized control trials compared the incidence of hypotension using remimazolam and other anesthetics. Among the studies, only one article was found for prolonged sedation in the ICU patients. Mechanically ventilated ICU patients received remimazolam (n = 30) or propofol (n = 30) to maintain deep sedation, defined as a Richmond Agitation and Sedation Scale (RASS) score of −  4 to − 5, in a pilot study [23]. The SOFA score was 7.0 [interquartile range: 4.0–8.0] in the remimazolam group, and 7.5 [5.0–9.0] in the propofol group. During the infusion for 48.0 [21.5–48.0] h in the remimazolam group, and for 28.0 [23.5–48.0] h in the propofol group, the average study drug infusion rate was 0.60 [0.45–1.07] or 2.53 [1.94–2.94], respectively. Remifentanil was co-administered at 6.0 [6.0–6.3] or 6.0 [5.7–6.1] µg/kg/h, respectively. Hypotension (systolic blood pressure < 80 mmHg or diastolic blood pressure < 50 mmHg) occurred in 53.3% or 60.0%, respectively. No bradycardia was observed in all patients. In another study examining geriatric patients ≥ 80 yr, remimazolam at 12 mg/kg/h or propofol at 90 mg/kg/h until loss of consciousness was given as an induction agent with co-administration of remifentanil at 0.25 µg/kg/min, followed by sevoflurane 1.5% and remifentanil at 0.05 µg/kg/min for anesthesia maintenance [24]. The incidence of hypotension (mean arterial pressure [MAP] < 65 mmHg) from the initiation of anesthesia until 3 min after tracheal intubation (72.1% in the remimazolam group and 72.7% in the propofol group), and the time course of blood pressure, were comparable between the groups. In another study examining high-risk patients with ASA-PS 3 or 4, who were anesthetized under adequate anesthesia level throughout the surgery using remimazolam (n = 270) or propofol (n = 95), hypotension (MAP < 65 mmHg) and noradrenaline bolus and/or infusion from the start of an anesthetic until 15 min after skin incision were more frequent under propofol anesthesia [25].

These studies indicated that remimazolam can cause hypotension with high frequency. Currently, it is still unclear how much remimazolam is superior to other sedatives for maintaining an appropriate blood pressure during deep sedation in ICU patients.

### Adverse event: delayed emergence

Both short-time sedation using remimazolam for procedures such as bronchoscopy and prolonged sedation can be performed in the ICU. A short-time sedation would not cause a delayed emergence because only small doses of remimazolam are administered to patients, resulting in minimal drug accumulation. In contrast, prolonged sedation can cause a delayed emergence due to the accumulation of remimazolam. Delayed emergence may be a problem in patients who are expected to be discharged from the ICU or who require assessment of consciousness and symptoms.

To prevent delayed emergence after remimazolam infusion, one considers two possibilities: (1) avoidance of an excessive dose, and (2) usage of the reversal agent, flumazenil. To avoid an excessive dose, care providers repeatedly assess sedation and increase or decrease the remimazolam infusion rate as needed to maintain the appropriate sedation level. However, as is well known, a constant-rate infusion can alter a drug's effect. Another issue is the high occupancy ratio of remimazolam to benzodiazepine receptors [26]. This fact may make it difficult to assess excessive doses of remimazolam. Yet another issue is the use of flumazenil, which appears to be another option for preventing delayed emergence. However, the dose of flumazenil can cause re-sedation with an excessive dose of remimazolam [27].

### Relationship between infusion rate and concentration of remimazolam

**Fig. 6:**
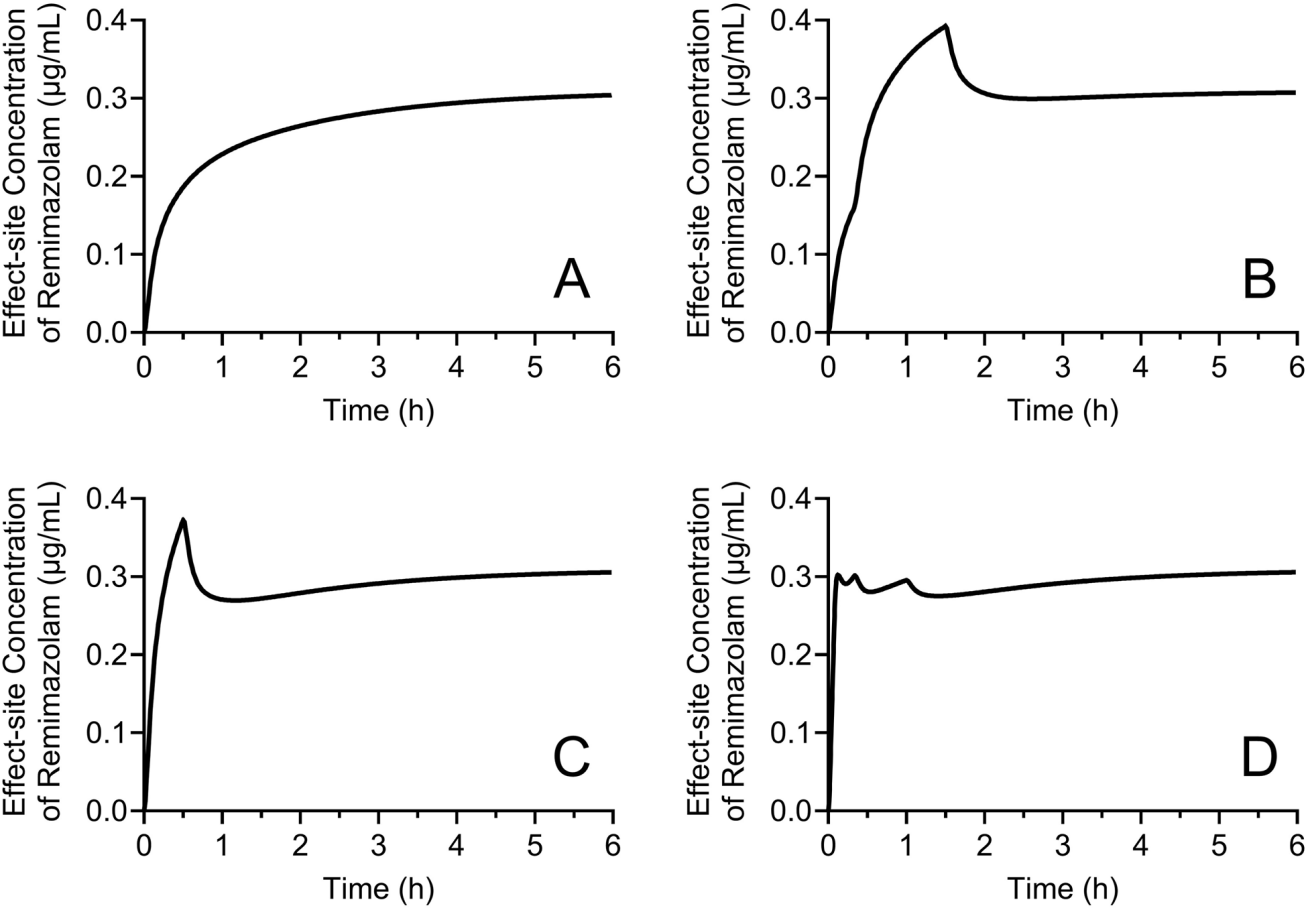
Time course of effect-site concentration of remimazolam during various infusion regimens in a male patient (60 years, American Society of Anesthesiologists physical status III, 60 kg, and 170 cm). The infusion regimens are: **A** 0.25 mg/kg/h for 6.0 h; **B** 0.25 mg/kg/h for 20 min, 0.40 mg/kg/h for 70 min, and 0.25 mg/kg/h for 4.5 h; **C** 0.5 mg/kg/h for 0.5 h, and 0.25 mg/kg/h for 5.5 h; **D** 1 mg/kg/h for 5 min, 0.4 mg/kg/h for 15 min, 0.3 mg/kg/h for 40 min, and 0.25 mg/kg/h for 5.0 h. The final infusion rate of all regimens is 0.25 mg/kg/h

As remimazolam is short-acting, one may assume steady state is reached shortly after the start of a constant-rate infusion or after a change in the dose rate. However, remimazolam concentration can change for several tens of minutes during a constant-rate infusion. Fig. 6 shows the time course of remimazolam Ce during various infusion regimens using the Masui remimazolam pharmacokinetic model [18, 19]. These figures indicate that remimazolam Ce can increase or decrease during a constant rate infusion.

When adjusting the remimazolam effect at a desired level, remimazolam concentration should be titrated. However, since actual remimazolam concentration cannot be measured at the bedside, care providers should adjust the remimazolam dose rate to achieve a desired effect level. As remimazolam Ce can change for several tens of minutes during a constant-rate infusion, care providers should take this phenomenon into account when assessing the relationship between the remimazolam dose rate and its effect. Such precautions would be taken when administering propofol. Note that the dose rate can be used in the same way as the remimazolam concentration during steady state when assessing the dose rate and remimazolam effect.

Pharmacokinetic simulation is also helpful for assessing the relationship between remimazolam concentration and effect and for controlling the dose rate, as it provides current and subsequent remimazolam Ce.

### Required concentration and dosing strategy of remimazolam in critically Ill patients in the ICU

Various dosing strategies are required for sedation in critically ill patients in the ICU because light, moderate, or deep sedation is necessary in young, middle-aged, or elderly patients. The following are ideas for dosing strategies for starting and maintenance doses up to > 24 h in a typical critically ill patient, the range of remimazolam infusion rates, and titration to adjust sedation level. Dosing strategies depend on the patient characteristics, such as not only age, which influences the pharmacodynamics of remimazolam, but also sex, obesity, and ASA-PS, which influence the pharmacokinetics of remimazolam (See the later paragraph named ‘Male/Female, Obesity, and ASA-PS Influence Infusion Rate’). Care providers should consider changing the infusion rate for remimazolam sedation. Given limited evidence for ICU sedation in children, this review focuses on the required concentrations of remimazolam and proposes dosing strategies for remimazolam sedation in adults based on published articles.

### Required concentrations of remimazolam for light/moderate/deep sedation

A previous article has shown the remimazolam concentrations for various sedation levels in healthy male volunteers aged between 20 and 38 years: median 0.337 [range 0.268–0.430] µg/mL for the modified Observer’s Assessment of Alertness and Sedation score (MOAA/S) [28] of 4, 0.481 [0.368–0.588] µg/mL for the MOAA/S of 3, 0.506 [0.387–0.615] µg/mL for the MOAA/S of 2, and 0.640 [0.403–1.384] µg/mL for the MOAA/S of 1 [29]. Another article has shown that the effective dose for loss of consciousness in a patient aged 70 or 45 years is approximately half or three-quarters of that in a patient aged 20 years [30]. Additionally, concomitant analgesic administration may reduce the required remimazolam concentration [31]. Based on these articles, the theoretical initial target concentration of remimazolam may be 0.1 µg/mL for light sedation, 0.2 µg/mL for moderate sedation, and 0.3 µg/mL for moderate sedation in elderly patients (70 years), and 1.5 or 2 times those concentrations in middle-aged (45 years) or young adult (20 years) patients.

### Proposal for a dosing strategy of remimazolam in ICU sedation

Based on the theoretical required concentration of remimazolam, a dosing strategy for ICU sedation can be established (Table 1). The infusion rate of remimazolam for deep sedation is equal to or lower than that for general anesthesia in high-risk patients [32]. There is individual variation in the pharmacodynamic effect of remimazolam, and concomitant administration of opioids may decrease the required concentrations of remimazolam. Therefore, dose adjustment is required based on the assessment of the remimazolam effect and the required remimazolam concentration in each patient. Note that this strategy was developed theoretically, not based on data from ICU patients. Further studies are warranted to determine appropriate sedation doses of remimazolam in the ICU.

**Table 1:** Proposal for dosing strategies of remimazolam in critically ill patients for ICU sedation

Basic dosing regimen to maintain the remimazolam concentration of 0.1 µg/mL			
Males	Initial dose		
	0.2 mg/kg/h for 15 min		
	Following doses		
	0.10 mg/kg/h for 45 min		
	0.09 mg/kg/h for next 60 min		
	0.08 mg/kg/h afterwards		
Females	Increase the dose rates by 10% compare to males		
**Target concentration for each sedation level**			
	Elderly (70 yr)	Middle-aged (45 yr)	Young adult (20 yr)
Light sedation	0.05–0.10 µg/mL	0.10–0.15 µg/mL	0.15–0.20 µg/mL
Moderate sedation	Twice the concentration for light sedation		
Deep sedation	Three times the concentration for light sedation		
**Dosing regimen**			
	Elderly (70 yr)	Middle-aged (45 yr)	Young adult (20 yr)
Light sedation	0.5–1.0 times the basic dosing regimen	1.0–1.5 times the basic dosing regimen	1.5–2.0 times the basic dosing regimen
Moderate sedation	1.0–2.0 times the basic dosing regimen	2.0–3.0 times the basic dosing regimen	3.0–4.0 times the basic dosing regimen
Deep sedation	1.5–3.0 times the basic dosing regimen	3.0–4.5 times the basic dosing regimen	4.5–6.0 times the basic dosing regimen
**Dosing adjustment**			
For increment or decrement to adjust the effect for the desired sedation level			
Typically, a 10–25% change in the infusion rate			
For specific patients			
Obesity: a 20% decrease in obese patients with a body mass index of 30 kg/m^2^			
Low-risk patient: a 5–10% increase			

### Tools and models for pharmacokinetic simulation

Pharmacokinetic software such as TIVAtrainer (https://www.tivatrainer.com), AnesSimulator (https://www.ut-anes.org/anessimulator/), and StanpumpR (https://stanpumpr.io) can simulate remimazolam concentration. For pharmacokinetic simulation, several pharmacokinetic models have been published for adults [18, 19, 29, 33–35] and children [35, 36].

### High receptor occupancy ratio and assessment of excessive doses

A published article has shown a theoretical occupancy ratio of remimazolam to benzodiazepine receptors [26]. In a virtual male patient (55 years old, American Society of Anesthesiologists physical status III, 170 cm, and 70 kg), remimazolam was infused to maintain a remimazolam Ce of approximately 1.0 µg/mL for 360 min. The occupancy ratio of remimazolam was 86% at the end of the infusion with the infusion rate of 0.7 mg/kg/h. This infusion rate is within the range to maintain a deep sedation level (RASS score of − 4 to −5) in the ICU patients, which was 0.60 [interquartile range: 0.45–1.07] in a randomized controlled trial [23]. Additionally, benzodiazepines do not directly activate the GABA_A_ receptor. [21, 22]. These suggest that an excessive dose of remimazolam might have little impact on clinical observations such as blood pressure, electroencephalographic waveforms, and electroencephalographic index, e.g., BIS value. If this speculation is correct, the remimazolam effect should be carefully assessed during long-term sedation, as remimazolam Ce might increase due to accumulation or changes in pharmacokinetics resulting from the patient's physical condition.

### Adverse event: delirium

A benzodiazepine, midazolam, is a risk factor for subsequent delirium after sedation in ICU patients [37]. Therefore, one may question whether remimazolam is also a risk factor for delirium. Recently, one meta-analysis, including six randomized controlled trials (RCTs) (n = 1107), concluded that the incidence of postoperative delirium (POD) did not differ between remimazolam and propofol groups [38]. Note that in five RCTs, propofol or remimazolam was used for both anesthesia induction and maintenance, whereas in one RCT, propofol or remimazolam was used only for induction and desflurane was used for maintenance.

Another meta-analysis, including 29 trials in which remimazolam was administered only for induction, only for maintenance, or both, found that POD occurred in 5% (95% confidence interval: 3–7%) of patients [39]. The incidences of POD concerning the patient characteristics were 1% (95% confidence interval: 0–1) for ASA-PS I or II, 19% (15–23%) for ASA-PS III-IV, 11% (3–9%) for children < 18 years, 1% (0–2%) for adults between 18 and 60 years, and 18% (4–13%) for elderly > 60 years. Additionally, the authors concluded that the higher remimazolam dose (> 0.3 mg/kg for induction, and > 1 mg/kg/h for maintenance) was linked to the lowest POD incidences, and the moderate remimazolam dose (0.2–0.3 mg/kg for induction, and 0.5–1.0 mg/kg/h for maintenance) was linked to the highest POD incidences. However, it is unclear whether an infusion rate > 1 mg/kg/h for anesthesia maintenance is better for other patients, as the infusion rates were not randomized in the collected data or meta-analysis.

### Specific population: male/female, obesity, and Asa-Ps influence infusion rate

The steady state concentration (C_ss_) during a constant rate infusion can be calculated as:

$$C_{ss} \, = \,\frac{Infusion\,Rate}{{CL}}$$

where C_ss_ is the steady state concentration of the drug (µg/mL), Infusion Rate is the remimazolam infusion rate (mg/min), and CL (L/min) is total clearance. When applying the Masui remimazolam pharmacokinetic model developed using 662 subject data from clinical trials [18], the equation is transformed to:

$$C_{ss} \, = \,\frac{Infusion\,Rate}{{(1.03\, + \,0.146\,\,Sex\, - \,0.184\,ASA)\,\,(ABW/67.3)^{0.75} }}$$

where C_ss_ is the steady state remimazolam concentration of the drug (µg/mL), Sex is 0 for male and 1 for female, ASA is 0 for ASA-PS I or II and 1 for ASA-PS III or IV, and ABW (adjusted body weight) is calculated as IBW + 0.4 (TBW − IBW), where TBW is total body weight (kg), and IBW is ideal body weight calculated as 45.4 + 0.89 (Height (cm) − 152.4) + 4.5 (1 − Sex). Accordingly, sex, body weight, and ASA-PS influence the steady state concentration of remimazolam. For example, regarding sex, in a patient who is 175 cm, 70 kg, ASA-PS I, and male or female, the C_ss_ are 0.330 µg/mL and 0.298 µg/mL, respectively, during a 0.3 mg/kg/h infusion; the female C_ss_ is 10% lower than the male C_ss_. For another example, regarding obese, in a patient who is 175 cm, male, ASA-PS I, and 70 kg, 92 kg, or 123 kg (body mass index, BMI: 23, 30, or 40 kg/m^2^, respectively), the C_ss_ are 0.330, 0.397, or 0.475 µg/mL, respectively, during a 0.3 mg/kg/h infusion; the C_ss_ for BMI 30 or 40 is 20% or 44% higher, respectively, than that for BMI 23. For yet another example, regarding ASA-PS, in a patient who is 175 cm, 70 kg, female, and ASA-PS I or III, the C_ss_ are 0.298 or 0.353 µg/mL, respectively, during a 0.3 mg/kg/h infusion; the C_ss_ for ASA-PS III or IV is 19% higher than that for ASA-PS I or II. The infusion rate of remimazolam should be titrated based on patient characteristics.

The equation to calculate the remimazolam C_ss_ was created with the Masui pharmacokinetic model for remimazolam [18], developed using the blood samples up to approximately 12 h after the initiation of remimazolam infusion from the volunteers and general anesthesia patients. Therefore, external validity is required to confirm whether this equation is appropriate for prolonged sedation > 12 h. A study showed the remimazolam concentration during prolonged ICU sedation (n = 10) at a rate of 0.1 mg/kg/h. The mean remimazolam concentration was 0.11 µg/mL during 24–48 h (n = 10), 0.12 µg/mL during 48–72 h (n = 9), 0.14 µg/mL during 72–96 h (n = 6), and 1.1 µg/mL during 96–120 h (n = 4) [40]. These are similar values to the calculated C_ss_: 1.4 µg/mL for an ASA-PS III male, 1.2 µg/mL for an ASA-PS III female, 1.1 µg/mL for an ASA-PS II male, 1.0 µg/mL for an ASA-PS II female. These results suggest that the above equation for the C_ss_ works well.

### Specific population: adult patients undergoing cardiac surgery or cardiovascular catheter procedure, or with hypertension

Several published RCTs focused on intraoperative hypotension. In patients undergoing transcatheter aortic valve implantation, intraoperative blood pressure was comparable between remimazolam (n = 28) and sevoflurane (n = 28) anesthesia [41]. The average norepinephrine dose differed by − 8.1 ng/kg/min (95% confidence interval:−  22 to 6.1) in the remimazolam versus sevoflurane anesthesia groups, without statistical significance. At the same time, perfusion index (P = 0.03) and regional cerebral oxygen saturation (P = 0.03) were significantly lower during surgery under remimazolam anesthesia. In patients undergoing catheter ablation, hypotension occurred 30% under remimazolam (n = 47) anesthesia and 59% under desflurane anesthesia (n = 49) [42]. The incidence of continuous vasopressor infusion was 19% versus 90%, respectively (P < 0.001). In hypotensive patients undergoing general, urological, or gynecological surgery, hypotensive episodes were fewer under remimazolam anesthesia (2 [0–3], median [interquartile range]) (n = 61) than propofol anesthesia (3 [1–5], P = 0.003) (n = 61) with an increase in intraoperative heart rate [43]. Incidence of norepinephrine boluses (1 [0–3] vs. 3 [1–5], P = 0.001) and total dose (8 μg [0–24] vs. 24 μg [8–40], p < 0.001) were lower.

Other published RCTs focused on postinduction hypotension under general anesthesia. In 65–75-year-old patients with hypertension, whose blood pressure was controlled below 160/90 mmHg, anesthesia induction with remimazolam 0.2 mg/kg (n = 30) resulted in higher blood pressure, higher heart rate, higher cardiac index, and lower systemic vascular resistance than induction with propofol 1.5 mg/kg (n = 30) (P < 0.001) [44]. In patients undergoing cardiac surgery, a continuous infusion of remimazolam at 6 mg/kg/h (n = 20), a single bolus of remimazolam 0.1 mg/kg (n = 20), and 0.2 mg/kg (n = 20) were given to compare the efficacy and hemodynamic stability during anesthesia induction [45]. The authors concluded that 0.2 mg/kg resulted in the shortest time to loss of consciousness without differences in blood pressure or heart rate compared with the other groups. In patients undergoing coronary artery bypass grafting, the incidence of postinduction hypotension was examined after a remimazolam 6 mg/kg/h infusion until loss of consciousness (n = 71) or a bolus of etomidate 0.3 mg/kg (n = 73) [46]. Postinduction hypotension with MAP < 65 mmHg occurred 36% in the remimazolam group and 25% in the etomidate group (P = 0.046), and postinduction hypertension with systolic arterial pressure > 140 mmHg did 36% in the remimazolam group and 58% in the etomidate group (P < 0.001).

Although the incidence of intraoperative hypotension is influenced by various factors, such as anesthetic agents, co-administered drugs, and patient characteristics, remimazolam appears to be associated with lower incidences of intraoperative hypotension than other agents, such as propofol and inhalation anesthetics, but not etomidate. The regimen of remimazolam administration would also influence the occurrence of postinduction and intraoperative hypotension.

### Specific population: children

Remimazolam has not been approved for children, but remimazolam would be desired especially for deep sedation for children in the ICU because propofol might cause propofol infusion syndrome [47, 48] and dexmedetomidine alone may be insufficient to achieve desired sedation [49]. Several publications have reported RCTs for general anesthesia in children. For induction of anesthesia in healthy patients with ASA-PS I or II, a single bolus is effective, with estimated doses of 0.45–0.60 mg/kg for 1–6 years and 0.35–0.45 mg/kg for 6–12 years [50]. These doses are 1.5–2 times or 3–4 times higher than the induction doses of young adults or elderly adults [30]. Another study investigated the safety and efficacy of remimazolam compared with propofol for general anesthesia in children aged 3–6 years with ASA-PS I or II [51]. All patients were successfully induced loss of consciousness using remimazolam 0.3–0.4 mg/kg (n = 140) or propofol 2.5–3.5 mg/kg (n = 47) and successfully maintained general anesthesia using remimazolam 2.0 mg/kg/h (1.8–2.2 [1.2–2.9]: interquartile range [range]) in 139 patients (99%) or propofol 8.4 mg/kg/h (6.9–9.6 [5.3–11.2]) in 46 patients with concurrent administration of remifentanil at 0.21 µg/kg/min (0.17–0.25 [0.10–0.49]) or 0.20 µg/kg/min (0.17–0.33 [0.12–0.40]), respectively, without a rescue anesthetic dose nor study drug discontinuation. Major adverse events, which occurred in ≥ 3% patients, were bradycardia (heart rate < 70 /min for > 5 min; 9%) and emergence delirium (4%) after remimazolam anesthesia, and bradycardia (26%), injection site pain (15%), emergence delirium (4%), nausea (11%), and vomiting (9%). These studies suggest that remimazolam might be an alternative for sedation in pediatric patients in the ICU. However, both studies examined healthy surgical patients with ASA-PS I or II. Although remimazolam can be used in critically ill patients with ASA-PS III [52], further investigation is warranted. Note that CSDTs in children are longer than those in adults [43], so a longer time will be necessary for emergence after termination of the remimazolam infusion.

### Specific population: patients with hepatic impairment

As mentioned above, remimazolam is metabolized by carboxylesterase 1 in humans, mainly in the liver. therefore, hepatic impairment appears to reduce remimazolam metabolism, leading to higher remimazolam levels compared to healthy patients. A pharmacokinetic analysis revealed that patients with Child-Pugh scores ≥ 9 (moderate or severe hepatic impairment) had altered remimazolam pharmacokinetics and reduced total clearance in severe hepatic impairment (Child-Pugh scores ≥ 10) [53], while the maximum observed concentration after a single dose of 0.1 mg/kg was not influenced by hepatic function. A case report described prolonged recovery (> 2 h) after 0.3 mg/kg remimazolam for anesthesia induction, followed by 0.8% sevoflurane for anesthesia maintenance, in a patient with severe hepatic impairment (Child-Pugh C) [54]. This patient required 1 h to regain spontaneous breathing after the single bolus of remimazolam. Another case report described remimazolam anesthesia in a patient with Child-Pugh C [46]. Remimazolam was infused at 0.3 mg/kg/h for most of the time during surgery, with BIS monitoring. As the patient remained unconscious after recovery of spontaneous respiration for 25 min, flumazenil was given, resulting in recovery of consciousness without re-sleeping. The infusion rate should be reduced in such patients for ICU sedation, similar to other sedatives such as dexmedetomidine and propofol.

### When suspecting benzodiazepine tolerance before and during sedation

Currently, it is unclear whether remimazolam should be avoided for ICU sedation in patients with suspected benzodiazepine tolerance. Theoretically, another sedative might be appropriate in such patients. However, remimazolam might be beneficial in some patients with hemodynamic instability. Clinical experiences indicate that remimazolam can be used for general anesthesia in some patients who were long-term benzodiazepine users. In such patients, caution may be necessary regarding the development of tolerance during remimazolam infusion.

### Is flumazenil helpful for recovery after remimazolam sedation?

Flumazenil administration may not achieve complete awakening without the risk of re-sedation, especially after a prolonged infusion and/or excessive dosing. After prolonged infusion, remimazolam accumulates in the body, resulting in a slow decay curve in its concentration. This phenomenon can be confirmed by CSDTs (Fig. 3). If the remimazolam concentration at the termination of its infusion is significantly higher than the concentration for awakening, a patient (male, 55 years, 70 kg, 170 cm, ASA-PS III) will take a long time to become awake. For example, when the remimazolam concentration is 1.0 µg/mL at the termination of a 12 h infusion, and the concentration for awakening is 0.3 µg/mL, the 70% decrement time indicates that it takes 49 min to become awake. In this case, flumazenil administration may cause re-sedation, as described in a published article [26]. As flumazenil is a competitive antagonist of benzodiazepines, a rapid decrease in flumazenil concentration after its dose can cause re-binding of remimazolam to the benzodiazepine receptors.

After a non-excessive dose of remimazolam, flumazenil may help to facilitate rapid recovery from remimazolam sedation. However, the following observations should be performed after a potentially excessive dose of remimazolam, especially after a prolonged infusion, which can cause slow remimazolam decay, or in elderly patients, whose effective dose is typically lower. Re-sedation or re-respiratory depression can occur several tens of minutes after flumazenil administration [26, 27]. The details of the pharmacological mechanism of re-sedation or re-respiratory depression have been explained elsewhere [26]. The usefulness of flumazenil in awakening patients is considered limited in clinical practice due to its potential risks.

Another perspective on the benefit of flumazenil reversal is the case of inducing sedation with airway difficulties. A large dose of flumazenil can reverse the remimazolam effect when a patient falls into a “cannot ventilate, cannot intubate” or “cannot intubate, can ventilate” situation [55, 56]. Additionally, flumazenil might be helpful when the confirmation of consciousness is necessary during remimazolam sedation.

### Clinical trials for approval for ICU sedation

A Phase II Clinical trial was conducted among ICU patients in Japan [9], in which higher plasma concentrations were observed in several patients than expected after prolonged infusion, resulting in prolonged recovery without significant unexpected adverse events. Accordingly, this trial was discontinued in 2013. Another phase II open-label investigator-initiated pilot study of remimazolam for sedation in critically ill patients (n = 30) has been published [57]. Remimazolam was initially administered at 0.25 mg/min, with adjustments as needed, up to a maximum rate of 1 mg/min. Fourteen patients reached the primary endpoint, which included achieving the targeted sedation level without the use of other hypnotic drugs and hemodynamic stability. Nine patients required another hypnotic (propofol [n = 6] or midazolam [n = 3]), and seven patients showed hemodynamic instability. Ten patients were sedated for > 48 h, and four received the maximum dose. Four adverse events were observed that led to remimazolam discontinuation (cardio-respiratory arrest [n = 1], vascular access site complication [n = 1], and hypotension [n = 2]). The plasma remimazolam concentration at the termination of infusion was > 5 µg/mL in two patients, suggesting potential tolerance to remimazolam.

## Conclusions

Remimazolam could be an alternative sedative because of its potential for lower incidence of hypotension, absence of propofol infusion syndrome, and the ability to achieve deep sedation, although it has not yet been approved. As remimazolam is not ultrashort-acting but short-acting, it accumulates after a prolonged infusion. The CSDT of remimazolam Ce helps understand the accumulation. Infusion rate should be titrated based on patient characteristics, including sex, body mass index, and ASA-PS, because these factors can alter the steady-state concentration of remimazolam by > 10%. However, there are several concerns for prolonged sedation, such as tolerance to benzodiazepines, including potential acute tolerance, and lower clearance in patients with severe hepatic impairment. Similar to other sedatives, concurrent use of additional hypnotics and analgesics may help maintain the desired sedation in ICU patients.

## Data Availability

No datasets were generated or analysed during the current study.

## Abbreviations

ABW: Adjusted body weight
ASA-PS: American society of anesthesiologists physical status
BMI: Body mass index
CSDT: Context-sensitive decrement time
CSHT: Context-sensitive half-time
C_ss_: Steady state concentration
GABA_A_: Gamma-aminobutyric acid type A
IBW: Ideal body weight
ICU: Intensive care unit
MAP: Mean arterial pressure
POD: Postoperative delirium
RCT: Randomized controlled trial
TBW: Total body weight
TCI: Target-controlled infusion
